# The lipid language of tuberculosis: *Mycobacterium tuberculosis* surface molecules in host interaction and drug resistance

**DOI:** 10.1128/mbio.03959-25

**Published:** 2026-02-02

**Authors:** Sandhya Krishnan Radhakrishnan, Varadharajan Sundaramurthy

**Affiliations:** 1 National Center for Biological Sciences, Bangalore, India; The Ohio State University, Columbus, Ohio, USA

**Keywords:** *Mycobacterium tuberculosis*, cell wall, host-pathogen interactions, multidrug resistance, *Mtb* lipids, pathogenesis

## Abstract

*Mycobacterium tuberculosis* (*Mtb*), the causative agent of tuberculosis (TB), is a uniquely successful pathogen due in large part to its complex lipid-rich cell envelope. Comprising nearly 40% of its dry weight, *Mtb* lipids—such as mycolic acids, phthiocerol dimycocerosates (PDIM), trehalose dimycolate (TDM), and sulfolipids (SLs)—play crucial roles in infection, immune evasion, intracellular persistence, granuloma formation, transmission, and drug resistance. These lipids modulate host-pathogen interactions by altering host membrane biophysics, hijacking phagosome maturation, and interfering with host immune pathways, including autophagy and inflammatory signaling. Upon inhalation, *Mtb* surface lipids inhibit pulmonary surfactant function and mask pathogen-associated molecular patterns, facilitating uptake by permissive macrophage subsets. Intracellularly, lipoglycans like mannose-capped lipoarabinomannan block phagolysosome fusion, while PDIM and TDM promote phagosomal escape and subversion of vesicular trafficking. Lipid-mediated modulation of autophagy pathways further enhances bacterial survival within host cells. In addition to shaping host immune responses, *Mtb* lipids orchestrate granuloma development and promote pathological features such as foam cell formation and caseation, which are central to transmission. Specifically, phenolic glycolipids and SLs stimulate neuronal pathways, triggering cough, thereby facilitating aerosol spread. Finally, the lipid-rich envelope acts as a formidable barrier to antibiotics, with resistance partly driven by the altered lipid composition and architecture in multidrug-resistant strains. Targeting lipid biosynthesis and transport pathways offers promising avenues for novel anti-TB therapies. This review highlights the multifaceted roles of *Mtb* lipids at the host-pathogen interface, recent technical advances enabling these insights, and emerging challenges in translating lipid biology into improved TB control.

## INTRODUCTION

*Mycobacterium tuberculosis* (*Mtb*), the etiologic agent of tuberculosis (TB), is the most successful human bacterial pathogen, estimated to infect a quarter of the global population, resulting in ~1.25 million deaths every year (1, 2). The extraordinary success of this pathogen is largely attributed to the unique and complex array of cell wall lipids that typify *Mtb*, such as mycolic acids (MAs), the exceptionally long-chain fatty acids (C60–C90), linked to the polysaccharide arabinogalactan (AG) or esterified to trehalose (3–5). *Mtb* lipids constitute ~40% of its dry weight, with the exact composition varying across strains and growth conditions (3–5). The complex chemical structure and organization of the cell wall account for the unique staining properties of mycobacteria, facilitating the diagnosis of infected specimens (4). At the host-pathogen interface, the *Mtb* lipids play critical roles in pathogenesis, wrestling with innate immune defenses and directing host-pathogen interactions by structurally altering the host membrane and acting as barriers against toxins and antibiotics. Studies in the last decade have established the role of *Mtb* lipids in deciding their niche. Phthiocerol dimycocerosates (PDIMs) mask the pathogen-associated molecular patterns (PAMPs) on mycobacteria and mediate the evasion of MyD88-dependent macrophage recruitment (6). In concert with this, the mycobacterial membrane phenolic glycolipids (PGLs) promote recruitment of permissive macrophages and have been postulated as the possible explanation for the ability of *Mtb* to colonize the relatively sterile lower respiratory tract, avoiding competition with commensal microbes residing in the upper respiratory tract (6, 7).

The co-evolutionary history of *Mtb* with humans has been successfully traced through the detection of stable mycobacterial lipid signatures found in ancient remains (8, 9). Studies of 9,000-year-old remains in Israel detected both GC-rich *Mtb* DNA and MAs, highlighting the protective role of cell wall lipids in DNA preservation. Similarly, evidence from human skeletons excavated from two sites in Syria confirmed human TB via lipid biomarkers (10, 11). Distinct lipid profiles also hint at species adaptation to various hosts and ecological niches. A 17,000-year-old bison skeleton revealed the presence of mycocerosates and PDIMs, characteristic of an *Mtb* infection (rather than *M. bovis*) (12). The observed transmission of *M. bovis* to humans and *Mtb* to cattle is evidence of ancient bidirectional transmission, before its transfer and establishment in humans (9, 12, 13). How the modifications in the cell envelope lipids have contributed to its adaptation across species and different stresses within its host is a subject of evolving understanding.

Eradication of TB remains an elusive dream, largely due to the surge of multidrug-resistant TB (MDR-TB) and extensively drug-resistant TB (1). *Mtb*’s lipid-rich cell envelope provides a formidable barrier against antibiotics (4), complement deposition, and oxidative stress (14, 15). Recent evidence indicates that drug-resistant (DR) *Mtb* strains may possess a more hydrophobic envelope, influencing infection and treatment outcomes (16, 17).

In this review, we discuss the multifaceted roles of *Mtb* cell surface lipids during infection. We detail their roles in promoting host cell entry, establishing and maintaining the intracellular niche, enabling adaptation to host environments by modulation of cellular functions, and their direct involvement in the development of drug resistance. We emphasize the significance of lipidomic diversity in these processes and highlight technical advances that are opening new questions.

## *Mtb* CELL WALL

**Fig 1:**
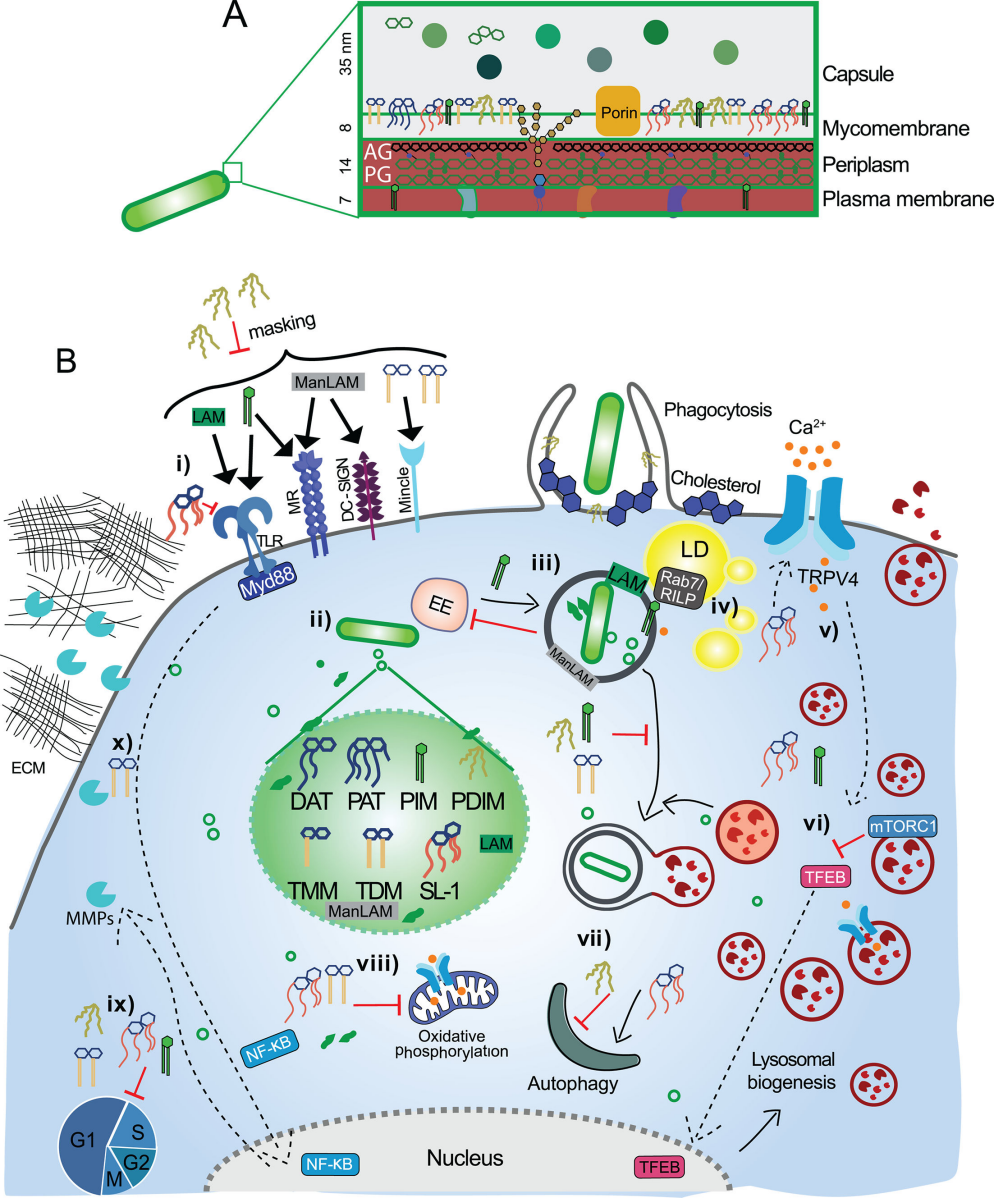
Overview of *Mtb*-phagocyte interactions depicting the diverse functions altered by *Mtb* lipids. (**A**) Mycobacterial cell wall architecture. (**B**) (i) Macrophage TLR2/1 recognizes Mtb lipoglycans LAM and PIMs and triggers the host immune response (40–42). SL-1 acts as a toll-like receptor 2 (TLR2) antagonist (43). Mannosylated mycobacterial lipoglycans ManLAM and related PIM_6_ are recognized via the mannose receptor (MR) on macrophages and dendritic cell-specific intracellular cell adhesion molecule-3-grabbing non-integrin (DC-SIGN) on dendritic cells, whose signaling cascades engage an anti-inflammatory response, enabling Mtb to evade the host immune surveillance (44, 45). Macrophage-inducible C-type lectin Mincle recognizes TDM through the trehalose motif and triggers pro-inflammatory cytokine production (46). PDIM physically masks mycobacterial PAMPs and prevents recognition by TLR and subsequent immune signaling cascades (6). PDIM also inserts into the host membrane in a cholesterol (raft)-dependent manner, thereby increasing the phagocytic uptake of *Mtb* (47). (ii) *Mtb* lipids can be released by passive shedding of the outer capsule and associated non-covalently attached lipids and actively by release of membrane vesicles (48). (iii) Inside the host cell, *Mtb* lipids are known to alter diverse functions: PDIM, TDM, and PIM are known to block the phagolysosome fusion and prevent the maturation of MCV. Lower-order PIMs and ManLAM have opposing effects on the fusion of MCV with early endosomes, but together coordinate the inhibition of phagosome maturation (49, 50). (iv) LAM and lower-order PIMs facilitate the association of MCV with lipid droplets (LDs) in the host via Rab7/Rab-interacting lysosomal protein (RILP) decked on LDs (51). (v) SL-1 activates transient receptor potential vanilloid 4 (TRPV4) to induce lysosome biogenesis and exocytosis in macrophages (52). (vi) Exogenous addition of *Mtb* whole-lipid extract, SL-1, and PIM_6_ induces lysosomal biogenesis via the mTORC1-TFEB pathway (53). (vii) SL-1 is also known to induce autophagy, while PDIM inhibits it (54, 55). (viii) SL-1 and TDM synergistically block host mitochondrial oxidative phosphorylation (56). (ix) Under the control of the *WhiB3* transcriptional regulator, *Mtb* lipids inhibit the host cell cycle (57). (x) TDM induces the production of MMPs in a MyD88-dependent manner via NF-κB activation and results in the degradation of the extracellular matrix (58). AG, arabinogalactan; PG, peptidoglycan; TLR, toll-like receptor; LAM, lipoarabinomannan; ManLAM, mannose-capped LAM; PIM, phosphatidylinositol mannoside; MR, mannose receptor; DC-SIGN, dendritic cell-specific intracellular cell adhesion molecule-3-grabbing non-integrin; TDM, trehalose dimycolate; PDIM, phthiocerol dimycocerosate; EE, early endosome; RILP, Rab-interacting lysosomal protein; mTORC1, mechanistic target of rapamycin complex 1; TFEB, transcription factor EB; MMP, matrix metalloproteinase; ECM, extracellular matrix.

Biochemical fractionation (18) and cryo-electron microscopy (19, 20) have allowed detailed characterization of the complex, thick, and waxy *Mtb* cell envelope. This multilayered structure consists of—moving from inside to outside—the cytoplasmic membrane (7 nm), a periplasmic space (14 nm) that consists of peptidoglycan (PG) covalently linked to the AG complex, which in turn is covalently bound to MAs in the outer mycomembrane (8 nm), and finally the capsule (35 nm) (21) (Fig. 1A). The cytoplasmic inner membrane primarily contains classical phospholipids such as phosphatidylinositol, phosphatidylethanolamine, and cardiolipin. In addition, they are rich in phosphatidyl-*myo*-inositol mannosides (PIMs), with one to six mannose residues attached to the inositol headgroup, based on which they are categorized as lower-order (PIM_1–4_) or higher-order PIMs (PIM_5_ and PIM_6_) (22). The less abundant PIM_2_ (22) resides in the inner leaflet of the inner membrane, while PIM_6_ occupies the outer leaflet (23). PIMs can get further acylated and facilitate tight packing, making the membrane impermeable to many drugs (23). Their hyperglycosylated derivatives, such as lipomannan and mannose-capped lipoarabinomannan (ManLAM), are anchored on the plasma membrane (PM) by their phosphatidylinositol anchor and extend outward through the periplasmic space and cell wall (24). The terminal arabinan motif determines the unique structure of ManLAM for each mycobacterial species (25). The PG layer is composed of a glycan backbone with repeating units of *N*-acetylglucosamine and *N*-acetylmuramic acid linked by β1→4 bonds, cross-linked by short peptide side chains. Importantly, PG from *Mtb* and *M. smegmatis* (*Msm*) contains both *N*-acetylmuramic acid and *N*-glycolylmuramic acid, which are absent in other mycobacteria (26, 27), and increases the resistance of the PG layer to host lysozyme (28). AG is mainly composed of arabinose and galactose sugars. A critical defining feature of the *Mtb* cell wall is its outer mycomembrane, within which a covalently linked mycolyl–arabinogalactan–peptidoglycan core forms the inner leaflet: here, MAs of three major classes (α-, methoxy-, and keto-mycolates) are esterified to AG. The outer leaflet is intercalated noncovalently with diverse lipids and glycolipids, including MA esters of trehalose, trehalose monomycolate (TMM) and trehalose dimycolate (TDM), as well as non-mycolate lipids such as PDIM, sulfolipid (SL), diacyl and penta-acyl trehalose (DAT and PAT, respectively), higher-order PIMs, LM, and lipoarabinomannan (LAM); free MA may also be detected at low abundance (29, 30). PDIM and structurally related PGL contain a lipid core with two esterified mycocerosate side chains, which are heterogeneous with respect to their lipid chain lengths (31, 32). DAT and PAT are features specifically associated with virulent isolates of *Mtb* (4, 33, 34)*,* while SLs are exclusively found in *Mtb* (35). The outer capsule, which appears as an electron-transparent zone surrounding the outer mycomembrane in conventional electron microscopy, mostly comprises sugars like α-D-glucan, D-mannan, and arabino-D-mannan, and proteins (36). Collectively, the *Mtb* envelope comprises an array of complex vertically stratified lipids with varied polarities—including glycolipids, aminolipids, phospholipids, phosphoglycolipids, SLs, and PGL. For a comprehensive view on the organization, biosynthesis, and regulation of the *Mtb* cell wall, readers are referred to other reviews (37–39). Here, we focus specifically on how *Mtb* lipids modulate host-pathogen interactions at the interface.

## MODULATION OF HOST FUNCTIONS BY *Mtb* LIPIDS FOR THE ESTABLISHMENT OF RESIDENCE IN THE HOST

This section explores the multifaceted roles of *Mtb* lipids throughout the infection cycle—from the initial pulmonary encounter to intracellular adaptation and eventual transmission—highlighting how these dynamic lipid-mediated processes underpin *Mtb*’s persistence, spread, and evasion of the host immunity.

### The early encounter: *Mtb* lipids and pulmonary surfactant

Upon entry into the lungs, *Mtb* encounters the pulmonary surfactant, a complex mixture of lipids (90%) and proteins (10%) secreted by alveolar type II epithelial cells (59, 60). TDM and MAs inhibit the activity of surfactant phospholipids in a model lung surfactant monolayer (61). Similarly, total lipids extracted from *Mtb* inhibited the surfactant activity of lavage bovine lung surfactant and calf lung surfactant extracts (62). Specifically, while TMM and TDM demonstrated *in vitro* surfactant inhibitory effects, both lower- and higher-order PIMs (a mixture of the abundant PIM_2_ and PIM_6_) did not (62). Conversely, human alveolar lining fluid and hydrolases from lung tissue significantly modify the *Mtb* cell wall, reducing the surface exposure of ManLAM and TDM, leading to a 60%–80% decrease in *Mtb* association with macrophages and intracellular growth (63). Transcriptomic analysis of *Mtb* upon exposure to calf lung surfactant showed upregulation of PDIM biosynthesis genes, potentially reflecting adaptive preconditioning for macrophage interaction (64). When tested at physiological concentrations, the hydrolases of alveolar lining fluid caused fragmentation of the *Mtb* cell wall; these fragments were effective in controlling *Mtb* infection in human macrophages in an IL-10-dependent manner (65). The surfactant-mediated modulations, with implications for alveolar macrophage (AM) activation, lung pathology, and transmission, highlight the complexity of early interactions, though the underlying biophysical mechanisms remain to be elucidated (66, 67).

### Recognition of *Mtb* lipids by host macrophages and roles in invasion

Several *Mtb* lipids orchestrate the establishment of an intracellular niche by mediating recognition, facilitating host cell entry, subverting host immune defenses, blocking phagosome maturation, and promoting escape into the cytosol (68–70). A diverse array of host surface receptors recognizes *Mtb* surface lipids as PAMPs, although PDIMs partially mask these ligands (Fig. 1B) (6). Beyond intracellular survival, different *Mtb* lipids modulate the host immune response and cell death pathways to promote bacterial persistence and dissemination (71, 72).

During infection, PDIM is transferred from the *Mtb* envelope to the host cell membrane (73). The conical shape of PDIM enhances phagocytosis (47), and the affinity of long methyl-branched chains to host cholesterol causes significant alterations to the host PM ordering (74) (Fig. 1B). The changes increase the PM fluidity, promoting efficient receptor-mediated phagocytosis via complement receptor 3 (75). Similarly, PGL-1 induces complement receptor 3-mediated phagocytosis (76).

Several *Mtb* surface lipids interact with diverse host immune receptors, leading to varied outcomes, as summarized in Table 1. TDM is directly recognized by C-type Mincle and the scavenger receptor macrophage receptor with collagenous structure (MARCO) (46, 77) (Fig. 1B). The binding of TDM to MARCO activates the TLR2 signaling cascade, ultimately inducing the production of nitric oxide (NO) and pro-inflammatory cytokines like tumor necrosis factor (TNF) (77). Lower-order PIMs also act as TLR2 agonists, triggering the activation of NF-κB and subsequent TNF production (78) (Fig. 1B). Similarly, lipomannan also functions as a TLR2 agonist, which specifically promotes the synthesis of IL-12 and induces apoptosis in host macrophages (79). In contrast, ManLAM and higher-order PIMs dampen inflammation; they are recognized by Dectin-2 and the MR on macrophages, as well as DC-SIGN on dendritic cells (22, 44, 80) (Fig. 1B). Apart from these agonists, PGL and SL-1 act as TLR2 antagonists, which dampen the crucial NF-κB activation (43, 76), illustrating the nuanced and multilayered mechanisms of immune modulation.

**TABLE 1:** Recognition of *Mtb* surface lipids by host receptors and modulation of host cellular processes

*Mtb* cell surface lipid	Host pattern recognition receptor	Impact on the host cell	Reference(s)
TDM	Mincle and MARCO	The TLR2 signaling cascade is activated upon binding to MARCO. Induces NO and proinflammatory cytokines like TNF	(46, 77)
PIMs	TLR2 agonist	NF-κB activation and TNF production	(78)
LM	TLR2 agonist	IL-12 production and apoptosis induction	(79)
ManLAM and higher-order PIMs	Dectin-2 and MR in macrophages; DC-SIGN on dendritic cells	Proinflammatory response inhibition and inhibition of IFN-γ and IL-12 production	(22, 44, 80)
PGL	TLR2 antagonist	Dampens TLR2 signaling and inhibits NF-κB signaling	(76)
SL-1	TLR2 antagonist	Inhibits NF-κB signaling	(43)

The entry of *Mtb* into host cells, particularly AMs, is significantly mediated by engagement of MRs by mannosylated lipids such as ManLAM and higher-order PIMs (22). ManLAM from virulent *Mtb* was the first identified ligand that binds MRs in macrophages (81). The terminal mannosyl units of ManLAM trigger actin remodeling and promote phagocytosis into AMs (82). Interestingly, while the higher-order PIMs bind MRs, resulting in inhibition of phagolysosome fusion, lower-order PIMs associate poorly with MRs (22). Furthermore, the acylation states of PIMs also influenced the association with MR. Thus, mannosylated lipids, ManLAM, and higher-order PIMs preferentially bind MRs depending on the degree of acylation and terminal mannose caps to modulate their intracellular fate (22). The degree of mannose capping and lipid acylation fine-tunes these interactions and intracellular outcomes, highlighting a targeted bacterial strategy to evade immune killing.

### *Mtb* surface lipids orchestrate intracellular survival and organelle homeostasis

Within the infected cell, several *Mtb* lipid effectors play significant, often redundant or overlapping roles in defining the intracellular niche (83). *Mtb* resides in a replicative phagosomal niche resembling the early endosome and prevents maturation of the *Mtb*-containing phagosome and fusion with degradative lysosomes (69, 70, 84–87). This early endosome-like vacuole allows the bacteria to acquire nutrients from recycling endosomes (83, 88, 89). The abundant *Mtb* lipoglycan, ManLAM, significantly affects phagosome maturation. Specifically, the engagement of MR by ManLAM significantly limits the increase in cytosolic Ca^2+^, which prevents activation of phosphatidylinositol 3-kinase and thus production of phosphatidyl inositol 3-phosphate (PI3P) (90). The glycophosphatidyl inositol (GPI) anchor of ManLAM inserts into host membrane rafts (91) and alters lipid membrane domains and cholesterol-sphingomyelin ratios in simulated phagosome membranes (92, 93). Interestingly, lower-order PIMs, which are the precursor of ManLAM, also integrate with host membranes (48), but enhance the homotypic fusion of early endosomes and the fusion between phagosomes and early endosomes, thus providing access to necessary nutrients (49) (Fig. 1B). Together, ManLAM and lower-order PIMs enable *Mtb* survival within host phagosomes by disparate, yet complementary actions: ManLAM inhibits selective recruitment of effectors that promote delivery of late endosomal/lysosomal constituents to *Mtb*-containing phagosomes, while lower-order PIMs stimulate fusion with early endosomes, allowing for nutrient acquisition, bypassing ManLAM-mediated inhibition of early endosome recruitment (49) (Fig. 1B). Interestingly, the structurally related PIM_6_, which also carries a GPI anchor, competitively inhibited the incorporation of ManLAM into human monocyte-derived macrophage (hMDM) membrane rafts and abrogated the effects of ManLAM on phagosomal maturation (93). Higher-order PIMs (PIM_5_ and PIM_6_), recognized by MRs, inhibit the recruitment of early endosomal antigen 1 to phagosomal membranes (94) by blocking the PI3P pathway-dependent delivery of lysosomal hydrolases from the trans-Golgi network to mycobacterial phagosomes, essentially arresting phagosome maturation (50). In addition to disrupting the phagosome maturation, LAM and lower-order PIMs facilitate the association of *Mtb*-containing vacuole with LDs in the host via Rab7/RILP decked on LDs (51), allowing exchange between the phagosome and LDs (Fig. 1B). Overall, the *Mtb* lipoglycans ManLAM and PIMs manipulate host phagosome maturation by blocking the lysosomal delivery and enhancing early endosome fusion, respectively, thereby creating a protective environment for the bacilli (Fig. 1B). Further evidence for host phagolysosomal manipulation comes from the lipophilic small molecule, 1-tuberculosinyladenosine (1-TbAd)—a terpene nucleoside abundant in TB clinical strains—which selectively accumulates in acidic compartments, causing lysosomal swelling and neutralization of the pH (95). Although producing 1-TbAd is metabolically expensive, its ability to remodel *Mtb* phagolysosomes to protect the pathogen from acid stress could help escape acid-mediated killing, leading to its natural evolution as a phagolysosome disruptor (95).

TDM is a key player in mediating host cell trafficking events. TDM-depleted *Mtb* were readily delivered to acidic compartments in mouse macrophages, while exogenous addition of TDM restored normal phagosome maturation arrest response, highlighting its specific role (96, 97) (Fig. 1B). Further, mutant *Mtb* lacking the enzyme *fbpA*, necessary for the transfer of mycolic acids to trehalose to synthesize TDM, showed enhanced fusion of mycobacteria-containing phagosomes with late endosomes in macrophages (98). Notably, *Mtb*Δ*fbpA* was more immunogenic in macrophages and dendritic cells due to its enhanced susceptibility to oxidants and phagosome maturation (97, 98). The molecular mechanism of its inhibitory action on phagosome maturation has been elucidated recently using a clickable, photoaffinity TDM probe (84). This probe captured the interaction of TDM with several soluble *N*-ethylmaleimide-sensitive factor attachment (SNARE) proteins, which normally promote fusion with other endosomes or lysosomes by complexing with vesicle-associated membrane proteins (VAMP) 7 or VAMP8. Crucially, TDM induced a non-canonical complexation with VAMP2 at the expense of VAMP8, thereby inhibiting phagosome maturation and promoting intracellular *Mtb* growth. This illustrates how a bacterial glycolipid can interfere with SNARE function, providing a striking example of an *Mtb* lipid–host protein interaction that subverts a fundamental host pathway to aid pathogen survival (84).

*Mtb* lipids play a key role in the escape of the bacilli from the phagosome to the cytosol, with a mutant lacking the PDIM transporter, Δ*mmpL7,* showing impaired escape (Fig. 1B) (69). Conversely, *Mtb* deficient for *Rv3167c,* a transcriptional repressor of the PDIM operon, exhibits higher levels of PDIM than the wild-type and shows increased escape into the cytosol, inducing more necrosis. Deleting the transporter *mmpL7* in the Δ*Rv3167c* background confirmed that PDIM leads to increased phagosomal escape and necrosis induction as a consequence of the increased number of cytosolic bacteria (69). Importantly, PDIM works synergistically with the ESX-1 substrate, EsxA, to enable phagosomal escape (69, 99). A study using an *Mtb* transposon mutant library to define functional relationships during macrophage growth and cytokine responses identified that mutants defective in ESX-1 and PDIM clustered together (100). The production and export of PDIM are vital for the coordinated secretion of ESX-1 substrates and downstream induction of type I interferon (IFN) response in macrophages (100). PDIM could thus play a novel role as a direct facilitator of ESX-1 substrate secretion for phagosome permeabilization (100). The concerted action of several *Mtb* lipids is thus required to sustain the intracellular niche, and we speculate that regulated shedding of these surface lipids, together with possible cell type- and time-dependent differences in this process, could introduce substantial heterogeneity in *Mtb* intracellular niches, with direct consequences for pathogenesis and drug susceptibility.

Xenophagy is a selective form of autophagy that delivers microbes to LC3-positive, double-membrane autophagolysosomes. While the role of autophagy in *Mtb* pathogenesis control is heavily debated (101–105), emerging work strongly implicates mycobacterial surface lipids in regulating these autophagic pathways. Total *Mtb* lipids increase both autophagy and mechanistic target of rapamycin (mTOR) signaling, suggesting that they induce autophagy in an mTOR-independent manner (106). SL-1 activates membrane-associated autophagy signaling through actin cytoskeletal remodeling (107). Strikingly, the sulfate group and fatty acids at 6- and 6’-positions are essential for the host membrane raft interactions as well as the autophagic response (108) (Fig. 1B). PDIM confers resistance to classical autophagy and inhibits LC3-associated phagocytosis by preventing the recruitment of NADPH oxidase in mice (54). The PDIM-overproducing Δ*Rv3167c* mutant also exhibited increased induction of autophagy (69). In contrast, *Mtb*Δ*mmpL7,* deficient in PDIM, showed reduced xenophagy, attributed to the requirement of PDIM for ESX-1-mediated phagosomal membrane damage. During acute infection, PDIM was required for survival within non-AMs in an autophagy-dependent manner but was dispensable in AMs (54), showing the cell type-specific role for PDIM in autophagy restriction. Additionally, PDIM but not SLs limit the acidification of LC3-positive *Mtb* compartments (109), though purified SLs modulate phagosome acidification by distinct mechanisms (53, 110). Interestingly, mutant *Mtb* lacking both PDIM and SL-1 promoted autophagy activation via MyD88-dependent signaling (109), while SL-1 limits this pathway by acting as a competitive TLR2 antagonist (43). This complex interplay between *Mtb* lipids, with PDIM and SL-1 exerting opposing effects on autophagy (Fig. 1B), underscores the distinct and sophisticated regulatory roles of individual lipids in modulating the host autophagic pathway. Emerging CLEM and FIB-SEM studies showing partial engulfment of *Mtb*-containing phagosomes by autophagosomes (111, 112) suggest spatial heterogeneity in surface lipid composition may orchestrate highly localized recruitment of autophagy machinery to discrete phagosomal microdomains in infected cells. The notion that *Mtb* lipids can spatially choreograph autophagy invites a re-examination of the long-held view of this pathway as a uniform, cell-wide process. A detailed understanding of the underlying molecular mechanisms and host targets could identify potentially novel forms of autophagy modulation of such large cargo and possibly novel strategies for therapeutic modulation of autophagy.

While these studies define lipid-mediated localized alterations aiding intracellular *Mtb* survival, emerging studies point to global rewiring of organelle function during *Mtb* infection. Exogenous addition of SL-1 increases cellular lysosomal content and acidification through the mTORC1 (mTOR complex 1)-transcription factor EB (TFEB) axis and accelerates the trafficking of phagocytic cargo such as *E. coli* to lysosomes (53). Recently, TRPV4, a non-selective mechanosensitive Ca^2+^ channel, was identified as upstream of TFEB in the SL-1-dependent global elevation of lysosomal content (Fig. 1B). Interestingly, TRPV4 activation, which causes calcium influx, promoted lysosomal expansion even in the absence of SL-1, suggesting that SL-1 might be exploiting this newly identified pathway to rewire host lysosomes during *Mtb* infection (52). In an infection context, the *Mtb* mutant lacking SL-1 biosynthetic machinery (Δ*pks2*) showed attenuated lysosomal rewiring, reduced delivery to lysosomes, and higher intracellular survival (53). In line with these findings, an *Mtb* strain overexpressing SL-1 showed enhanced delivery to lysosomes (110), as did beads coated with Ac_4_SL (a major form of SL-1) (110), underscoring the effect of SL-1 on phagosome maturation. However, Passemar et al. reported that in the absence of both PDIM and SL-1, *Mtb* was trafficked more efficiently to lysosomes than the wild-type (113), suggesting that the loss of PDIM could have dominant changes in the *Mtb* cell wall (113). In addition to SL-1, total *Mtb* lipids and PIM_6_ also result in lysosomal expansion (53) (Fig. 1B), reiterating the concerted effects of multiple lipids in promoting cellular effects. These observations establish a direct correlation between cellular lysosomal content and phagosome maturation kinetics—both of which are remarkably modulated by *Mtb* surface lipids. Yet the cellular logic underlying the lipid-mediated lysosomal expansion remains unresolved. One interpretation could be that host cells recognize *Mtb* lipids as PAMPs or metabolic cues that trigger TFEB-dependent upregulation of lysosomal biogenesis as a containment strategy. Alternatively, given the emerging roles of lysosomes as signaling hubs that coordinate transcription, antigen presentation, immune cell migration, and cell death pathways (114–116), active *Mtb*-driven modulation of lysosomal quantity and function could provide the pathogen with a powerful lever to manipulate these interconnected host processes in a context-dependent manner, effectively converting the host organellar networks into pathogenic effectors. However, whether *Mtb* lipids actively alter the quality of induced lysosomes in terms of their enzymatic composition, pH gradients, lipid content, or spatial distribution remains unknown and represents an underexplored dimension of *Mtb*-lipid mediated mechanisms to subvert host cell homeostasis.

### *Mtb* lipids induce biophysical changes and cell cycle arrest in host cells

The insertion of *Mtb* lipids into the host PM during bacterial entry induces dramatic biophysical changes. PDIM, as well as sulfoglycolipids, physically reorganize the host PM domains, especially at the fluid regions, as demonstrated by live polarization imaging of THP-1 macrophages treated with SL-1 (107). Notably, SL-1 affects the host membrane order, fluidity, and the actin cytoskeleton below the host membrane without affecting the viability of the cell. [1] DM and PDIM increase membrane stiffness (47, 55, 107, 108), while SL-1 and PGL-1 induced membrane softening and enhanced membrane fluidity in THP-1 macrophage membranes, without affecting lipid diffusion (55, 117). How these lipid-induced biophysical changes in the host PM translate into alterations of cellular function during infection remains largely unexplored.

WhiB3 is a major virulence factor and redox regulator, enabling *Mtb* to adapt to the phagosomal environment by remodeling the cell wall and modulating metabolism (57, 118). *WhiB3*-controlled lipids are critical for establishing and maintaining persistent infections and may serve as targets for host-directed therapies (57). Interestingly, *Mtb* infection inhibits macrophage G1/S cell cycle transition via polyketides under the control of *Mtb WhiB3* (57). Specifically, the authors identified that *Mtb* total lipids, as well as several purified individual lipids including SL-1, PDIM, mycolic acid methyl esters, lower-order PIMs (PIM_1_ and PIM_2_), and TDM, arrested the host cell cycle at the G0/G1 stage (57) (Fig. 1B). This finding aligns with that of an earlier study that reported cell cycle arrest upon infection of cultured bronchial airway epithelial cells with *M. bovis* Bacillus Calmette Guérin (BCG) (119). While the mechanistic details underlying *Mtb* lipid-mediated cell cycle blockade remain incompletely understood, recent evidence reveals a compelling functional connection between host cell cycle phase and *Mtb* phenotypic heterogeneity (120). Intracellular *Mtb* residing in G2-phase cells experiences markedly higher oxidative stress compared to bacteria in G1-phase cells. This observation raises an intriguing hypothesis: by imposing G1/S arrest through lipid-mediated signaling, *Mtb* may actively sculpt its intracellular niche to maintain a less stressful reductive microenvironment conducive to its replication (121, 122). Such precise environmental tuning through surface lipids exemplifies the sophisticated multilayered strategy by which *Mtb* surface lipids orchestrate host cell biology at cellular and organellar levels to establish and sustain a permissive intracellular niche.

### *Mtb* lipids as virulence factors and immune modulators

Recent classifications group the *Mycobacterium tuberculosis* complex into nine major evolutionary lineages (L1–L9), comprising four *Mtb sensu stricto* lineages (L1–L4) and five *M. africanum* lineages (L5–L9), linked to specific geographic human populations and broadly categorized as “ancient” and “modern” lineages (123–126). While there is wide variation in virulence among *Mtb* strains from different lineages, modern lineages typically elicit a weaker inflammatory response than ancient strains (127, 128). *Mtb* whole lipids extracted from East Asian/Beijing and Indo-Oceanic strains (modern and ancient) induced higher concentrations of proinflammatory cytokines like TNF and IL-1β from mouse bone marrow-derived macrophages (BMDMs). However, the broader bacterial cell wall lipid profile, especially the PDIMs, was highly conserved within each lineage (129). Intriguingly, phenolphthiocerol dimycocerosate, PGL, produced by the Indo-Oceanic strain, did not influence the secretion of TNF and IL-1β, but a related PGL in the Beijing strain was sufficient to alter cytokine expression, suggesting lineage-specific lipid alterations (130). Similarly, ManLAM showed subtle structural variations in the mannose capping and acyl modifications between ancient and modern lineages (131). Specifically, the ancient lineage strains contained a newly identified acetoxy/hydroxybutyrate modification, while they were absent or at very low levels in the modern lineages (131). While these studies show a role for *Mtb* lipids in modulating inflammatory responses, a clear correlation between lineage-specific distinctions in lipid profiles and virulence is not apparent (129, 130, 132–136). It is quite likely that subtler differences, such as distinct modifications of specific lipids, may play a predominant role. Better analytical methods to detect such changes and consistency in lipid preparation methods will help establish these standards.

Several *Mtb* lipids are considered virulence-associated due to their strong phenotypes during infection. Collectively, the *Mtb* virulence lipids can be classified into those essential for adaptation and survival (MAs and TDM), which are highly conserved across strains, factors that attenuate the host immune response (PDIMs) present in all clinical isolates of *Mtb,* and lastly, lipids that modulate host response (SL-1 and PGL), which show lineage-specific enrichment (56). The foundational identification of PDIM as a virulence lipid was based on studies showing that PDIM-deficient *Mtb* had reduced replication and tubercle formation (137). While PDIM was necessary for the multiplication of *Mtb* within infected mouse lungs, it was not essential for persistence (138) or for the inhibition of phagolysosome fusion in resting macrophages. However, PDIM protected *Mtb* from reactive nitrogen intermediates produced by activated macrophages (138). During infection of mice, PDIM shields *Mtb* from the host’s early innate response (2 weeks), independent of the MyD88-dependent mechanism involving IFN-γ, reactive nitrogen intermediates, and reactive oxygen species (139). The structurally related PGL (synthesized by the pks15/1 gene) also acts as a key virulence factor in certain lineages (140). Studies using mutants lacking the PGL biosynthetic machinery (Δ*pks15/1*) showed an inverse correlation between PGL production and pro-inflammatory mediators like TNF, IL-6, IL-12, and MCP-1 (136). Although PGL production actively suppresses the secretion of host cytokines and contributes to hypervirulence in mouse models, it requires synergy with other virulence factors to achieve this effect (141). The role of SL-1 in *Mtb* pathogenesis was first reported by Goren et al. in 1974 (137). Profiling the transcriptional response of human immature dendritic cells exposed to SL-1 and its synthetic analog showed that SL-1 did not modulate the classical inflammatory genes like TNF, MIP2, IL-10, and NF-κB, unlike TDM and ManLAM (142). Deleting sulfotransferase *stf0*, which initiates SL-1 biosynthesis, did not affect bacterial growth or membrane properties *in vitro* or in murine *in vivo* infections but reduced the *Mtb* survival in human macrophages, suggesting potential species-specific differences (142). Given that the levels of SL-1 are tightly controlled by its biosynthesis and cell wall localization (118, 143), further examination of the bacterial and environmental cues that regulate SL-1 biosynthesis, specifically in the context of infection, is necessary (142) to enable integration with the newly emerging functions such as lysosomal expansion and cough response, as detailed elsewhere in this review.

*Mtb* lipids also play a direct and critical role in the adaptive immune response. A landmark study by Beckman et al. demonstrated that MA could be presented to CD1b-restricted αβ+ T cells, marking the first instance of lipid antigen recognition by T cells (144). Subsequent studies reported the recognition of isoprenoid glycolipid antigens by CD1c T cells (145), emphasizing the role of mycobacterial lipids as immune activators. ManLAM is recognized by αβ+ T cells, and its successful (antigen) presentation requires internalization and endosomal acidification (146). Furthermore, diacylated sulfolipids (Ac_2_SL) stimulate CD1-restricted T cells (147), with their fatty acid chain methylation patterns and acylation sites on trehalose governing T cell recognition and activation (148). Interestingly, while fatty acyls are critical for T cell recognition, they are dispensable for the antagonistic activity of Ac_2_SL on TLR2 (43), illustrating how the structural complexity of *Mtb* lipids enables engagement with distinct host receptors to elicit divergent cellular outcomes. A comprehensive summary of mycobacterial lipid recognition by immune receptors is provided by Ishikawa et al. (149).

Deciphering the combined action of diverse *Mtb* lipids during infection remains a critical frontier. Gene deletion mutants have been essential to dissect lipid-specific roles but cannot fully resolve functional redundancy among lipids, especially long-chain fatty acids (4). Moreover, cooperative effects, such as the metabolic coupling between PDIM and SL-1 (149) and the synergistic inhibition of mitochondrial oxidative phosphorylation by SL-1 and TDM (56, 150) (Fig. 1B), are often obscured. Comprehensive lipidomic profiling and systematic studies of lipid combinations are therefore needed to isolate individual lipid contributions within the integrated mycobacterial lipidome.

### *Mtb* lipids in granuloma formation and organization

Experiments injecting purified *Mtb* lipids into animals demonstrated that granuloma-like lesions form even without live infection, highlighting the role of lipids in their formation. Lower- and higher-order PIMs (the abundant PIM_2_ and PIM_6_) act alike in eliciting a granulomatous response, with the lipidic part playing a crucial role in their activity. Structure-activity relationship studies revealed an absolute requirement for at least one fatty acyl chain to generate a granulomatous response and to recruit natural killer T cells, irrespective of the number, position, or length of acyl chains (150, 151).

Necrotic or caseating granulomas are characteristic lesions of active TB, crucial for successful transmission. When mice previously sensitized by a low-dose *Mtb* infection were injected with TDM intraperitoneally as an oil-water emulsion, caseating granulomas formed directly in the peritoneal cavity (152), suggesting that the immune response against TDM is central to caseation pathogenesis. The kinetics of immune cell recruitment to TDM-induced granulomatous lesions differ by mouse strain, with BALB/c mice showing greater natural killer and T-cell influx compared to the less responsive C3H/He mice (153). Intravenous injection of TDM into rabbits also induced prominent granulomas in the liver and lungs. However, injection with SL-1, which also possesses a trehalose moiety, did not induce granuloma formation (154). TDM also plays a critical role in hypersensitivity-type (T cell-dependent) as well as foreign body-type (T cell-independent) mechanisms of granulomatous inflammation (155, 156).

A hallmark of granulomas is the presence of lipid-rich macrophages, called foamy macrophages. In addition to inducing granulomas, TDM also induced the formation of foamy macrophages in mice and *in vitro* (157). Consistent with this, transcriptional profiling of caseous human pulmonary TB granulomas revealed high expression of genes related to lipid sequestration and metabolism (157). This evidence collectively implicates *Mtb* lipid-mediated dysregulation of host lipid metabolism in the progression of TB granuloma toward caseation (157). *Mtb* lipids are thus active architects of granulomatous pathology, scripting the lesions meant to contain the infection. Elucidating the molecular circuitry through which *Mtb* lipids orchestrate these complex inflammatory architectures remains a central challenge for understanding and ultimately controlling tuberculous pathology.

### *Mtb* lipids in transmission

*Mtb* lipids play central roles in manipulating diverse host processes to facilitate their dissemination from infected cells and eventually, from the infected individual. PGL activates the stimulator of interferon genes (STING) cytosolic sensing pathways in the infected AMs, leading to production of CCL2, which recruits CCR2^+^ monocytes toward the infected AM. Transient fusion between the infected AMs and CCR2^+^ monocytes enables bacterial transfer and subsequent dissemination throughout the host (158). TDM contributes to tissue breakdown and aids late-stage dissemination and transmission in a MyD88-dependent manner (58). TDM induces the MyD88-dependent production of several matrix metalloproteinases (MMPs), including MMP-8, 9, 12, 13, 14, and 19, indicating a shift toward an ECM-degrading phenotype in an *in vivo* granuloma model (58) (Fig. 1B).

Cough is the persistent hallmark symptom of TB and the central means of *Mtb* transmission from the infected individual. Based on the hypothesis that *Mtb* actively mediates its own transmission, Ruhr et al. tested the cough reflex stimulation by *Mtb* organic extract and found SL-1 as a key cough-triggering molecule that activated nociceptive neurons (71). *Mtb* extract, but not SL-1, also stimulates non-nociceptive human neurons but not mouse neurons, suggesting additional mechanisms in inducing cough, pointing to potential species-specific differences (159). A more recent study from the Shiloh group tested the hypothesis that lineage 2 strains of *Mtb*, which have higher virulence and transmission, may carry additional neuron-activating molecules that trigger cough (72). Purified PGL triggers the cough response in guinea pigs, and structure-activity relationship studies showed that the saccharide chain of PGL was sufficient for neuronal activation. Mechanistically, PGL activates neuronal signaling by stimulating the release of extracellular ATP via pannexin channels. A combination of SL-1 and PGL significantly triggered an increase in the intracellular calcium compared to either lipid alone (72), demonstrating combinatorial effects of *Mtb* lipids in aiding transmission. While strain- or lineage-specific differences in lipid composition account for differential transmission, the large number of glycolipids, including DAT, TAT, PAT, SL-1, TMM, TDM, PDIM, and PIM, can have a variable distribution on the *Mtb* surface across different life cycle stages of the same strain and drive the surface hydrophobicity in favor of their airborne transmission (160). Although both the cough response and granuloma evolved as host protective mechanisms against infections (161–164), *Mtb* has leveraged them to facilitate its survival and enhance transmission.

### How do *Mtb* surface lipids access their diverse targets in the host?

*Mtb* surface lipids must exit the bacterial cell to access and manipulate various host targets, but the mechanisms governing their trafficking remain poorly understood. An intriguing possibility is the direct release of non-covalently attached cell wall lipids inside the infected cells (47, 48, 74). A pioneering study by Beatty et al. used a hydrazine-labeled *M. bovis* BCG strain to demonstrate the release and redistribution of cell wall origin lipids throughout the host cell, with a concentration on the endo-lysosomal organelles (48). In addition to this passive shedding, the release of mycobacterial vesicles budding from the bacterial surface has been observed in culture and within host phagosomes (165) (Fig. 1B). Partial characterization of the mycobacterial vesicles revealed that they are composed of *Mtb* proteins and lipids such as PIMs, LAM, PAT, and PGL (165). Strikingly, the released extracellular vesicles are enriched for LAMP-1 and mycobacterial lipids, suggesting exocytosis from the cell through calcium-dependent lysosomal exocytosis (166), which spread to uninfected “bystander” cells (48). *Mtb* lipids are thus not confined to the mycobacteria-containing vacuole of the infected cells; instead, they actively and passively traffic throughout the host cell, reaching other organelles, membranes, and even uninfected cells to exert their diverse functions. Recent advances in click chemistry have enabled the detection of an individual lipid (PDIM) and tracked its spreading during *M. marinum* infection of a zebrafish model (74). Investigating the precise nature of *Mtb* lipid shedding—such as compositional and temporal variations, potential regulatory mechanisms, and effects on different bystander cells—represents an area of fertile future research, with potential implications for understanding pathogenesis and identifying novel therapeutic targets. By escaping the bacterial envelope and traversing host membranes, *Mtb* lipids effectively blur the spatial boundaries between the pathogen and host and act as mobile effectors that rewire fundamental host processes such as trafficking, immunity, and metabolism across cellular landscapes.

### A note of caution

Mounting evidence shows that *Mtb* surface lipid composition is remarkably fluid, shifting with the culture medium used, growth phase, and processing conditions. Passage of *Mtb* through a narrow-gauge needle is a routine method to prepare single *Mtb* suspensions for infection assays. However, recent RNAseq analysis by Mittal et al. revealed that such suspensions introduce artifacts through alterations in cell wall lipids, including PDIM (167). These changes significantly affect macrophage inflammatory responses via TLR2 pathways and reduce intracellular survival in BMDMs. These findings underscore the sensitivity of non-covalently attached lipids like PDIM to experimental manipulations and emphasize their critical role in *Mtb* infectivity (167). Furthermore, *in vitro* culture of *Mtb* in the presence of surfactants like Tween-80, commonly used to prevent bacterial clumping, has been shown to cause PDIM loss. Mulholland et al. have suggested supplementing cultures with propionyl-CoA and avoiding Tween 80 to minimize the emergence of heterogeneous PDIM populations (168). Host cholesterol also serves as a key source of propionyl-CoA for the biosynthesis of PDIM and SL-1 via methylmalonoyl-CoA in the absence of sugar carbon sources (169). Biochemical analyses of *Mtb* grown on 7H11 agar revealed a significant decrease in the levels of ManLAM, LM, and AcPIM_6_ from day 14, while the abundance of lower-order PIMs increased between days 9 and 28 (170). In addition to these changes, Middlebrook 7H9 and 7H11 agar, commonly used for *Mtb* culture, contain supraphysiological concentrations of inorganic phosphate (Pi) (171). When cultured in Pi-starved conditions, *Mtb* utilizes alternate phosphate sources from the host and regulates gene expression necessary for survival (172, 173). Gray et al. observed that more than 50% of *Mtb* surface lipids were altered when cultured in Pi-free media and phospholipids were replaced with previously undetected phosphorus-free lipids, such as ornithine lipids and glucuronic acid-containing lipids (171). Interestingly, the abundance of TMM was higher, suggesting the remodeling of the lipidome extends beyond the PM, as TMM shuttles MA to the mycomembrane (171).

These data document the plasticity of *Mtb* surface lipid composition, while also highlighting a key assumption that the *in vitro* bacterium faithfully mimics its *in vivo* counterpart. The lipids that define *Mtb’s* interface with the host and govern its evasive power are also most vulnerable to common experimental procedures, including detergent washes, extended culture, and mechanical manipulation during single-cell preparation. Rigorous control of growth and handling conditions is essential, and the field urgently needs standardized lipidomic benchmarks or sentinel readouts that warn of compositional drift. Without such safeguards, studies of *Mtb*–host interaction risk compounding genuine lipid signatures during infection with artifacts associated with altered lipid composition.

## *Mtb* LIPIDS IN ANTI-TB DRUG ACCESSIBILITY AND DRUG TOLERANCE

The lipid-laden cell wall is a formidable barrier for the entry of drugs, contributing to the high degree of inherent resistance of *Mtb* to several antibiotics (174). Thus, drug accessibility through the hydrophobic cell wall is a significant consideration in anti-TB drug development, alongside other factors of acquired resistance such as mutations in the drug target, increased target expression, drug inactivation, and upregulation of efflux pumps (175–178).

DR *Mtb* clinical isolates show substantial changes in their lipid profiles, particularly in triacylglycerols, PIMs, and MAs (56, 179, 180), showing a correlation between lipid composition and drug resistance. PIMs were less abundant in DR strains than in drug-sensitive strains, with PIM_2_ levels being lower and PIM_6_ being undetectable in the resistant strains (179). Indeed, increased abundance of hallmark lipid species such as PDIMs, PGL, and MAs is thought to promote the acquired drug resistance phenotype (179).

**Fig 2:**
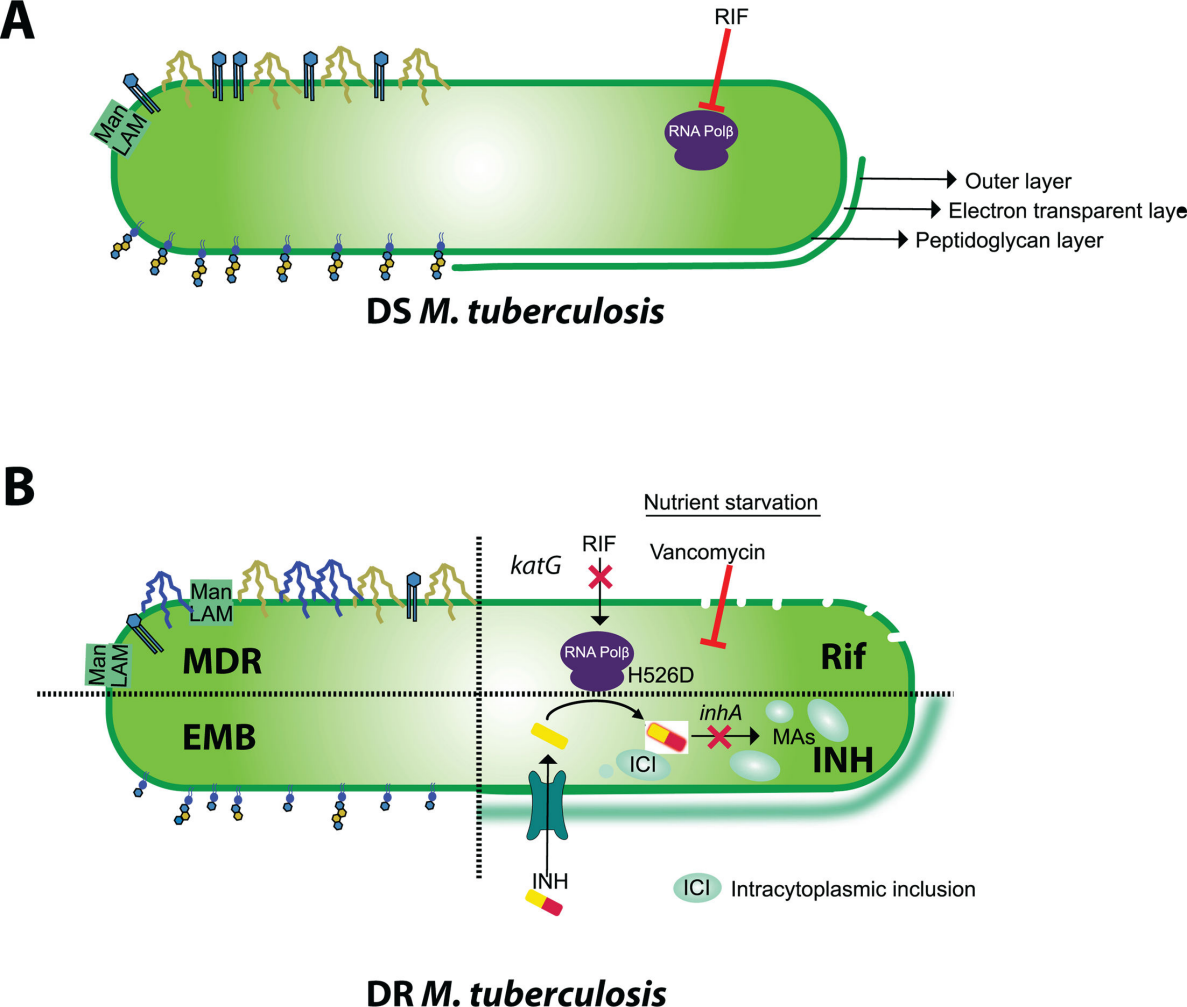
Lipid alterations in drug-resistant *Mtb*. This schematic illustrates the differences between (**A**) drug-susceptible (DS) and (**B**) drug-resistant (DR) *Mtb* clinical isolates as reported in the literature. MDR strains exhibited significant remodeling of their cell wall, such as increased hydrophobicity, partly due to the accumulation of PDIMs and PDIM variants (shown in blue, not present in DS strains), increased cell surface exposure of ManLAM, and decreased exposure of PIMs (16). The rifampicin-resistant *rpoB* H526D mutant showed increased cell wall permeability and was susceptible to killing by vancomycin in nutrient starvation conditions (187). Exposure to suboptimal concentrations of isoniazid or host-induced stresses led to thickening of the electron-transparent layer and formation of intracytoplasmic inclusions in clinical isolates (191). The ethambutol-resistant CSU20 strain was associated with structurally heterogeneous LAM variants, ranging from small, non-mannose-capped variants to fully capped variants, which are typically absent in the lab strain H37Rv (192). RIF, rifampicin; INH, isoniazid; ICI, intracytoplasmic inclusions; EMB, ethambutol.

The W-Beijing *Mtb* strains are associated with increased drug resistance (181). Infection with MDR W-*Mtb* strains induces type I IL-1 receptor 1 (IL-1R1) signaling pathway, driving the induction of IFN-β to reprogram macrophage metabolism toward aerobic glycolysis (182). The single-nucleotide polymorphism (SNP) in *Mtb* that reprograms host macrophage metabolism was mapped to the *Mtb* RNA polymerase-β H445Y (*rpoB*-H445Y); RNA Polβ is the canonical target of rifampicin (RIF) (183–185). Importantly, the *Mtb rpoB* SNP mutant overexpressed PDIM and bypassed the IL-1R1 signaling pathway for protective immunity. Furthermore, the abundance and composition of PDIMs in the MDR strains were found to impact macrophage metabolic rewiring (182). In line with this, proteomic analysis of the RIF-resistant, *Mtb rpoB* mutant revealed an upregulation of PDIM biosynthesis both in broth culture and during murine macrophage infection (186). Conversely, the commonly observed *rpoB* mutation H526D in clinical strains was associated with increased *Mtb* cell wall permeability and susceptibility to *in vitro* cell wall stresses and drugs under nutrient-deprived conditions (187) (Fig. 2). Nutrient starvation is an *in vivo* stress stimulus that elicits persistence and antibiotic tolerance in *Mtb* (188, 189). Predictably, the *Mtb rpoB* mutant H526D had higher susceptibility to cell wall-damaging drugs like vancomycin following nutrient starvation (187) (Fig. 2). The upregulation of PDIM precursors could represent a feedback mechanism to compensate for the apparent loss of PDIM in the *rpoB* mutants (187). Additionally, whole-genome sequencing of *Mtb* mutants from a transposon mutant screen with altered antibiotic tolerance revealed SNPs that abolished PDIM synthesis (190). These studies collectively suggest that PDIM acts as a catalyst of *Mtb* antibiotic tolerance triggered by nutrient starvation (182, 190).

Isoniazid (INH) is a prodrug that is intracellularly converted to isonicotinoyl, forming an isonicotinoyl-NAD^+^ adduct, which inhibits MA synthesis. The loss of MAs makes the mycobacteria susceptible to chemical damage and dehydration (193, 194). Upon INH treatment of the clinical isolates, the thickness of the cell envelope electron-transparent layer (usually composed of lipids like MAs) was reduced, coinciding with the accumulation of lipid inclusions (Fig. 2). Critically, *Mtb* exhibited reduced acid-fastness under these stressed conditions (191).

The EMB-resistant *Mtb* clinical isolate (CSU20) produces truncated non-mannose-capped structural variants of LAM (Fig. 2). Interestingly, LAM extracted from CSU20 was structurally and immunogenically closer to LAM from *M. leprae* than to the commonly used lab strain *Mtb* H37Rv (192). This suggests that the structural variants of arabinans in LAM could account for their *in vivo* immunological activity, and the unique LAM variant in *M. leprae* could indeed contribute to its reported natural resistance to EMB (195–198).

Further comparisons between DS and DR strains show that DR strains have a higher amount of hydrophobic PDIMs and a lower amount of hydrophilic PIMs (Fig. 2) (16). ManLAM surface exposure was also higher in DR strains. These hydrophobic DR strains were associated less with hMDMs, suggesting lower initial uptake but exhibited increased intracellular growth rates compared to DS strains (16). Furthermore, multiple PDIM alkyl forms unique to DR-*Mtb* strains were reported. In contrast, there was a significant decrease in the higher-order PIMs in MDR and pre-XDR-*Mtb* strains compared to DS strains, with no difference in the lower-order PIMs (16). However, these studies did not perform genomic analysis of the clinical isolates to pinpoint the origin of the observed differences in PDIM levels.

Beyond cell wall changes, the ability of *Mtb* to persist is linked to the internal lipid dynamics. Ultrastructural analysis using transmission electron microscopy of *Mtb* in the sputum of pulmonary TB patients displayed a triple-layered cell envelope and accumulation of intracytoplasmic lipid inclusions, unlike the *Mtb* H37Rv lab strain (191) (Fig. 2). Variations in the accumulation of intracytoplasmic lipid inclusions among clinical *Mtb* isolates were also observed in culture conditions. Importantly, the accumulation of lipid inclusions increased under stressed conditions such as oxidative, iron-deficient, and antibiotic-stressed (INH-treated) environments.

Overall, PDIM emerges as a central *Mtb* lipid promoting tolerance to multiple first-line anti-TB drugs. Targeting PDIM biosynthesis or function, particularly in combination with host-imposed stress, may represent a promising strategy to enhance drug efficacy and shorten TB treatment regimens.

## CONCLUSION AND PERSPECTIVES

The diverse and dynamic lipid repertoire of the *Mtb* cell surface is central to its pathogenesis, governing host immune responses, intracellular survival, granuloma architecture, transmission strategies, and drug tolerance. Understanding the precise biological roles of these lipids requires a nuanced, integrated approach, given their complex and concerted biological effects. To fully understand how the host environment influences *Mtb* lipid composition and, in turn, how these lipids modify host pathways, we must precisely elucidate their host targets and the logic of their concerted action in the infection context.

A crucial knowledge gap remains regarding the dynamic remodeling of the *Mtb* lipidome *in vivo* during various stages of infection and within different cell types and sub-cellular microenvironments. Current studies are largely limited to *in vitro* bulk measurements, despite evidence that environmental factors drastically impact virulence lipid production (199). Future research must focus on comprehensive lipid profiling under physiologically relevant stresses (hypoxia and oxidative stress) and achieve single bacterial resolution to capture the full spectrum of adaptive modifications and heterogeneity (121, 200).

Addressing these gaps is possible with emerging chemical biology and imaging tools. Novel chemical probes incorporating clickable groups are instrumental in tracing the spatiotemporal dynamics of mycomembrane construction and remodeling (74, 84, 201–203). New approaches that multiplex chemical tools with advanced imaging and genetics are offering unprecedented insights into *Mtb* metabolism and the deconvolution of host protein targets (204). Moreover, experimental setups that mimic *in vivo* conditions, such as the aerosol fluid model, have shown that genes critical for PDIM and SL-1 synthesis are essential for survival during transmission and adaptation to the alveolar conditions (205, 206). A crucial next step in the field is understanding how *Mtb* lipids are adapted to aerogenic transmission, aiding survival outside the host and facilitating infection using experimental models (207).

These advances affirm the sophisticated and dynamic roles of *Mtb* lipids in pathogenesis, positioning them as compelling targets for innovative diagnostics, biomarkers, therapeutics, and vaccines. The translational potential of disrupting *Mtb* lipid biosynthesis and transport pathways is profound, offering promising strategies to enhance existing drug regimens and tackle MDR-TB. Furthermore, lipid-based biomarkers hold great promise to improve diagnostic accuracy and enable precision patient stratification for host-directed therapies. By decoding the lipid language of TB, we can not only deepen our understanding of microbial pathogenesis but also pave the way for transformative clinical interventions. Realizing this potential will require continued multidisciplinary collaboration, rigorous standards, and the development of cutting-edge tools to harness lipid biology in the global fight against TB and move closer to its ultimate eradication.
